# Embryonic Development of the Bicuspid Aortic Valve

**DOI:** 10.3390/jcdd2040248

**Published:** 2015-10-02

**Authors:** Peter S. Martin, Benjamin Kloesel, Russell A. Norris, Mark Lindsay, David Milan, Simon C. Body

**Affiliations:** 1 Department of Anesthesiology, Perioperative and Pain Medicine, Brigham and Women’s Hospital, Harvard Medical School, 75 Francis St., Th724, Boston, MA 02115, USA; E-Mails: peter.martin001@umb.edu (P.S.M.); b.kloesel@gmx.net (B.K.); 2 Department of Regenerative Medicine and Cell Biology, Children’s Research Institute, Medical University of South Carolina, 173 Ashley St, Charleston, SC 29403, USA; E-Mail: norrisra@musc.edu; 3 Cardiovascular Research Center, Richard B. Simches Research Center, Massachusetts General Hospital, 185 Cambridge Street, Boston, MA 02114, USA; E-Mails: lindsay.mark@mgh.harvard.edu (M.L.); dmilan@mgh.harvard.edu (D.M.)

**Keywords:** bicuspid aortic valve, heart development, congenital heart disease, aortic valve, aortic stenosis, aortic incompetence

## Abstract

Bicuspid aortic valve (BAV) is the most common congenital valvular heart defect with an overall frequency of 0.5%–1.2%. BAVs result from abnormal aortic cusp formation during valvulogenesis, whereby adjacent cusps fuse into a single large cusp resulting in two, instead of the normal three, aortic cusps. Individuals with BAV are at increased risk for ascending aortic disease, aortic stenosis and coarctation of the aorta. The frequent occurrence of BAV and its anatomically discrete but frequent co-existing diseases leads us to suspect a common cellular origin. Although autosomal-dominant transmission of BAV has been observed in a few pedigrees, notably involving the gene *NOTCH1*, no single-gene model clearly explains BAV inheritance, implying a complex genetic model involving interacting genes. Several sequencing studies in patients with BAV have identified rare and uncommon mutations in genes of cardiac embryogenesis. But the extensive cell-cell signaling and multiple cellular origins involved in cardiac embryogenesis preclude simplistic explanations of this disease. In this review, we examine the series of events from cellular and transcriptional embryogenesis of the heart, to development of the aortic valve.

## 1. Introduction

Congenital heart disease (CHD) is the most frequently occurring birth defect among liveborn humans. Bicuspid aortic valve (BAV) in particular is the most common congenital valvular heart defect, having an overall frequency of 0.5%–1.2% and occurring more frequently in males and white individuals. BAVs result from abnormal aortic cusp formation during valvulogenesis, whereby adjacent cusps fuse into a single large cusp resulting in two, instead of the normal three, aortic cusps [1]. BAV is a strong risk factor for accelerated aortic valve disease, principally aortic stenosis, and ascending aortic aneurysm [2,3].

BAV occurs in several genetic syndromes, including Turner syndrome, and is associated with coarctation of the aorta, hypoplastic left heart syndrome, Holt-Oram syndrome, ventricular noncompaction and adult-onset aortopathy [1]. In addition, BAV occasionally demonstrates complex inheritance in large families without evidence of syndromic features, other congenital heart defects or a family history of CHD. Yet, some 10% of BAV patients have first-degree relatives with the condition or associated non-valvular abnormalities such as aortic coarctation, thoracic aortic aneurysms, mitral valve disease or ventricular septal defects [4].

Over the past few decades, much progress has been made in understanding the embryological development of the heart, including the contributions of various cell populations and the molecular biology of its very complex spatial and temporal anatomical development. Advances in the identification of transcription factors, signaling molecules and structural genes for heart development have assisted the discovery of genes causing CHD. Culprit genes have been identified using genomewide association studies (GWAS) or candidate gene sequencing [5,6,7], which have elucidated the etiology of some congenital heart defects, notably atrial and ventricular septal defects [8,9,10,11] and Tetralogy of Fallot [12,13,14]. However, the underlying pathology of most structural outflow tract (OFT) defects remains unknown because of the complexity of transcriptional and signaling mechanisms in the developing embryo and the numerous cell types involved in tract development. Thus far, for any single structural defect, numerous genes have been implicated, each in only a small percentage of patients. Similarly, there has been limited progress in defining the molecular etiology of BAV. Although autosomal-dominant transmission of this condition has been observed in some three-generation pedigrees, notably involving the gene *NOTCH1* [15,16], no single-gene model clearly explains BAV inheritance. Likewise, evidence for specific loci for a genetic etiology of sporadic BAV (wherein no first- or second-generation relatives with BAV can be identified) is limited.

In this review, we describe normal development of the aortic valve; the key cellular players; the specifics of cell-cell communication and migration; and pathways possibly involved in BAV development.

## 2. Progenitor Development of the Vertebrate Heart

The vertebrate heart has remarkable evolutionary conservation and structural similarity that includes divided pulmonary and systemic circulations, a conduction system and valves to create coordinated unidirectional flow at high pressure. These diverse structural and functional features of the vertebrate heart reflect its origination from cardiac neural crest (CNC) cells, which contribute to portions of the OFT and septum; the mesenchyme that comprises the first heart field (FHF) and second heart field (SHF); the endothelium, which provides growth factor signals and precursor cells for formation of the cardiac valves; and the proepicardium, which provides precursors for the coronary vasculature and mural valve leaflets [17,18]. Spatial and temporal integration of these various cell types is required for proper cardiac specification and anatomical morphology during embryogenesis.

### 2.1. Gastrulation in the Early Embryo

**Figure 1:**
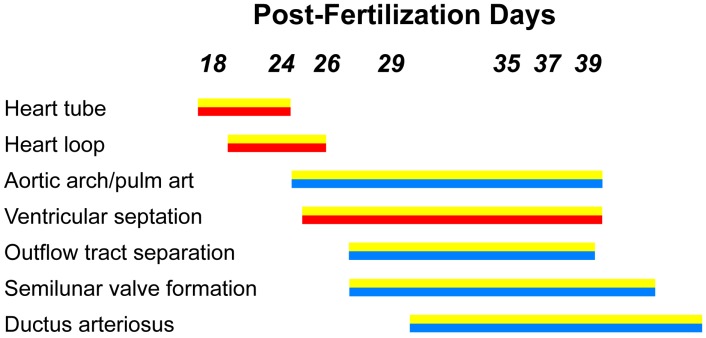
Timeline of outflow tract and semilunar valve development post-fertilization. The colors represent contributions to cardiac development from different cell populations. These contributions are from the first heart field (red), second heart field (yellow) and cardiac neural crest (blue). Modified from [19].

We start at gastrulation as specification of cardiac progenitor cells occurs by this time. After formation of the hollow sphere of cells known as the *blastocyst*, the three germ layers of the embryo (the ectoderm, mesoderm, and endoderm) are formed by development of the primitive streak (along the anterior-posterior axis) and gastrulation—the process of conversion of the spherical blastocyst to the complex and differentiated *gastrula* that forms the organs at about days 15–16 of human embryogenesis (Figure 1). Gastrulation requires cell polarity, axis specification and cell-type specification to establish a three-dimensional map of the embryo.

Embryonic progenitor cells derive from the epiblast on either side of the primitive streak. The process of forming three germ layers from the blastocyst requires epiblast migration and epithelial to mesenchymal transition (EMT) to form the mesoderm and endoderm. Finally, cell lineages of the heart, pharyngeal arches and vasculature begin to be defined. EMT is a process by which epithelial cells lose their cell polarity and cell-cell adhesion, and gain migratory and invasive properties to become mesenchymal stem cells, which are multipotent stromal cells that can differentiate into a variety of cell types. After undergoing EMT, mesodermal cells move cranio-laterally before converging medially while forming the lateral plate mesoderm (LPM) and other mesodermal derivatives. The LPM splits into the dorsal somatic and ventral splanchnic mesoderm. The dorsal somatic mesoderm gives rise to the body wall and other derivatives, while the ventral splanchnic mesoderm gives rise to all heart components mentioned earlier as well as other derivatives.

EMT is primarily mediated by transforming growth factor-β (TGF-β) signaling as well as Wnt (wingless-related MMTV integration sites), fibroblast growth factor (FGF) and the bone morphogenic pathway (BMP), amongst others. Expression of cardiac-specific transcription factors, such as the *GATA*, *MEF2*, *NK2*, *TBX* and *HAND* gene families, control cardiac cell fate and the morphogenesis of cardiac structures. Adjacent endodermal cells that are not destined to become cardiac precursors express many of these transcription factors and are paracrine controllers of development. Thus, complexity of the vertebrate heart results from spatial and temporal selectivity of a diverse range of regulatory proteins and regulatory elements on specific cell populations. This specificity in gene regulation probably underlies the restriction of many cardiac defects to specific anatomical regions of the heart [5].

### 2.2. Cardiac Mesoderm (FHF and SHF)

Mesodermal progenitor cells derived from the LPM arise from the cranial third of the primitive streak during early gastrulation. These mesodermal cells migrate in a cranial-lateral direction to become localized on either side of the primitive streak and undergo EMT to mesodermal cells, then proliferate and migrate in a semicircular fashion. Later, they coalesce in the midline to form the cardiac crescent.

**Figure 2:**
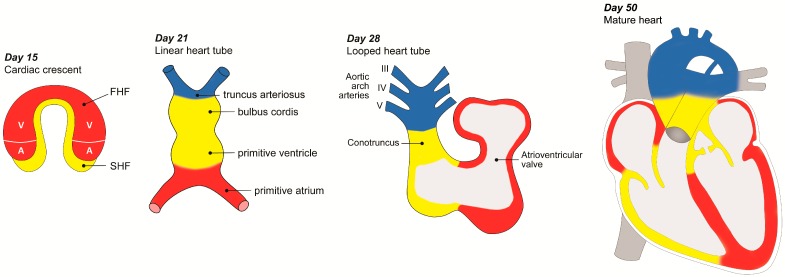
Stages of human cardiac development with color-coding of contributing cell populations, seen from a ventral view. These contributions are from the first heart field (red), second heart field (yellow) and cardiac neural crest (blue). By day 15 post-fertilization, the cardiac crescent is specified to form specific segments of the linear heart tube, which is patterned along the anteroposterior axis to form the looped and mature heart. Cardiac neural crest cells populate the aortic arch arteries (III, IV, and VI) and aortic sac (AS) that together contribute to specific segments of the mature aortic arch. Mesenchymal cells form the cardiac valves from the conotruncal (CT) and atrioventricular valve (AVV) segments. Abbreviations: A, atrium; V, ventricle; RV, right ventricle; LV, left ventricle; RA, right atrium; LA, left atrium; PA, pulmonary artery; Ao, aorta; DA, ductus arteriosus. Modified from [24].

The cellular fate of these cardiac mesodermal cells is initially uncommitted, but after migration, they become specified to differentiate into the cardiogenic mesoderm of the FHF and SHF at about day 18 of human embryogenesis (Figure 2). The FHF is formed from the cells that are first to differentiate from the cardiac crescent and are marked by the transcription factor *Hcn4*. The FHF gives rise to the early heart tube and, later, to much of the left ventricle, portions of both atria and some of the right ventricle [20].

The SHF is composed of undifferentiated progenitor cells from the medial splanchnic mesoderm adjacent to the pharyngeal endoderm. Many transcription factors have been implicated in the development of the SHF, including Mef2c, Tbx1, Islet-1 (Isl1), Hand2, Fgf8 and Fgf10 [20]. Once the rudimentary heart tube is created, cells from the SHF contribute to the OFT, giving rise to the smooth muscle of the aortic root, and join with more distal smooth muscle of the ascending aorta derived from neural crest cells. The SHF is also the source of the majority of the myocardium of the right ventricle, components of the interventricular septum, a small part of the left ventricle and the atria [21,22]. Specifically, the SHF cells reach the heart tube at both the arterial pole (anterior SHF) and venous pole (posterior SHF). At the arterial pole, the anterior SHF-derived mesoderm supplies the myocardium of the right ventricle up to the right side of the ventricular septum. The SHF also contributes to the semilunar valves and the walls of the great arteries. Progenitors of SHF cells are marked by the transcription factor *Isl1* [23].

### 2.3. Cardiac Neural Crest Cells

Neural crest cells form a wide variety of cells types, including nervous tissue, melanocytes, cartilage, bone, connective tissue and smooth muscle. CNC cells are a population of multipotent neural crest cells that originate from the dorsal neural tube at the interface of the ectoderm and neural ectoderm during embryogenesis. They migrate bilaterally into the ectoderm and endoderm of the pharyngeal arches as each arch develops and proliferate with the pharyngeal arteries to form the smooth muscle layer of the carotid artery (3rd pharyngeal arch), upper limb arteries and aortic arch (4th pharyngeal arch), and pulmonary arteries (6th pharyngeal arch).

A subpopulation of CNC cells continues caudal migration to the anterior domain of the SHF to form the OFT [25] at about day 22 in the human, where they contribute to the aorta, main pulmonary artery, and aortic-pulmonary septum via the truncal cushions [26]; valve formation via the endocardial cushions; and perhaps separation of the ventricular myocardium from the aorta and pulmonary artery [26]. The CNC cells at the base of the OFT lie in the medial walls of the arterial trunks, continuous proximally with the remnant of the aorto-pulmonary septum [27]. The 4th and 6th pharyngeal arches are connected to the proximal OFT by the aortic “sac”—a non-muscular intra-pericardial connection that becomes the ascending aorta by septation of these arches; rotation of the OFT enables connection of the septated arches to their respective ventricles [28]. Thus, septation of the elongated spiraling OFT occurs with fusion of the single aorto-pulmonary septum and enlarged endocardial cushions after significant CNC cell colonization of the truncal cushions.

CNC cells also provide signals that shape the developing heart. For example, they pattern the aortic arch, ventricular and atrial septae, and OFT [29]. Similar to requirements for the EMT process, Wnt, FGF and BMP are necessary for CNC cell migration and programming. Disruption of the endothelin, semaphorin, Wnt/β-catenin-Pitx2, Notch, TGF- β, BMP and Hedgehog pathways results in septal and OFT defects.

### 2.4. Myocardial and Endocardial Cell Contributions

The fate of cardiogenic mesoderm cells is shaped by their communication with the notochord and the pharyngeal endoderm of the embryo. The endocardium in particular plays a crucial role in several steps of the development of the early heart, including cell specification and guiding cell migration. It is thought that mammalian endocardial and myocardial cells are derived from EMT of a common multipotent progenitor within the cardiac mesoderm [30,31]. The endocardium plays two key roles during OFT morphogenesis: population and proliferation of the endocardial cushions, and regulation of OFT myocardial proliferation. In response to signaling from the conal OFT myocardium, the endocardium undergoes EMT to give rise to a population of mesenchymal cells that seed the initially acellular endocardial cushions, which then remodel into the arterial valves [32].

Signaling between CNC and SHF cells is required for OFT development [33,34], and removal of SHF progenitors results in OFT defects [35]. These observations show the importance of multiple extra-cardiac and cardiac cell lineages needed for OFT development. Migration of extra-cardiac lineages into the heart and OFT requires precise temporal and spatial regulation and signaling [36].

## 3. Development of the OFT

The OFT extends from ventricular myocardium to the margin of the pericardial cavity and is made up of the infundibulum (conus) nearer the right ventricle, and the proximal aorta and pulmonary artery (truncus) nearer the branchial arches [37,38]. The lumen of the OFT is continuous with that of the aortic sac of the pharyngeal arch arteries.

**Figure 3:**
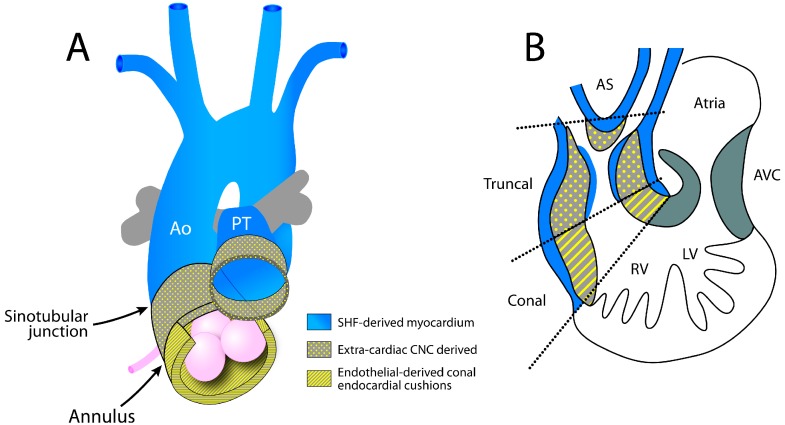
Genesis and cellular contributions to the outflow tract. Schematic shows the locations of outflow tract (OFT) colonization by the extra-cardiac cardiac neural crest (blue), vascular smooth muscle derived from the second heart field (dotted yellow) and the location of myocardium from derived from the second heart field (striped yellow). The aortic annulus or hinge region is formed where myocardial cells meet the vascular smooth muscle cells of the media of the aorta and pulmonary trunk and endothelial derived mesenchyme is the source of the fibroblastic annular tissue. The media of the aorta and pulmonary trunk is derived from secondary heart field proximally (dotted yellow) and the cardiac neural crest distally (blue). The interface between these populations is at the sinotubular junction. Abbreviations: Ao, aorta; AS, aortic sac; AVC, aorto-ventricular cushions; LV left ventricle; PT, pulmonary trunk; RV, right ventricle. Modified from [36] and [27]

The OFT is derived from CNC and FHF cells that give rise to endocardium and myocardium, as well as cells derived from the SHF, and from EMT of endothelial cells (Figure 3) [30,36,38]. Additionally, luminal endothelial cells of the OFT vessels undergo EMT to contribute to the endocardial cushions that, together with the migrating CNC cells, form the OFT septum [36]. The distal OFT originates from CNC-derived mesenchyme cells [39,40], whereas the proximal OFT originates from endocardial-derived mesenchyme [41]. During early embryogenesis, CNC cells migrate ventrally as mesenchymal cells to populate the distal OFT, where they merge with SHF and endocardial cells to form the aorto-pulmonary septum [39]. This septum divides the initially single embryonic OFT (also called truncus arteriosus) into aortic and pulmonary arteries, establishing separate systemic and pulmonary circulations.

Much of the development of the OFTs is confused by naming conventions of structures present during embryogenesis and by different interpretations of anatomical, histologic and lineage-tracing models in different species [42]. It also seems likely that some of the observations in quail-chick models are not applicable to primate embryogenesis and that mouse models offer better insight. Nonetheless, there are some well-established principles to cell origin and specification in the OFT.

### 3.1. Development of the Aorto-Pulmonary Trunk

Cells from the branchial mesenchyme, the paired 3rd/4th and 6th pharyngeal arteries, project into the pericardial cavity as an aortic sac. The 3rd and 4th pharyngeal arteries eventually form the aortic arch, while the initially paired 6th pharyngeal arteries fuse to form the pulmonary artery and ductus arteriosus. When the OFT is first formed, it has exclusively muscular walls that extend to the borders of the pericardial cavity, where the tract becomes continuous with the aortic sac. This sac is initially separated from the truncus but elongates to fuse with the distal truncus. Within the lumen, the endocardial jelly concentrates into pairs of facing cushions that continuously extend the length of the OFT, spiraling around one another as they run from the distal end of the right ventricle to the aortic sac. The cushions are proteoglycan-rich structures that eventually become populated by three mesenchymal cell populations: CNC-, SHF- and EMT-derived cells. The core of the protrusion is derived from the SHF, but it is covered by material derived from the neural crest [43].

**Figure 4:**
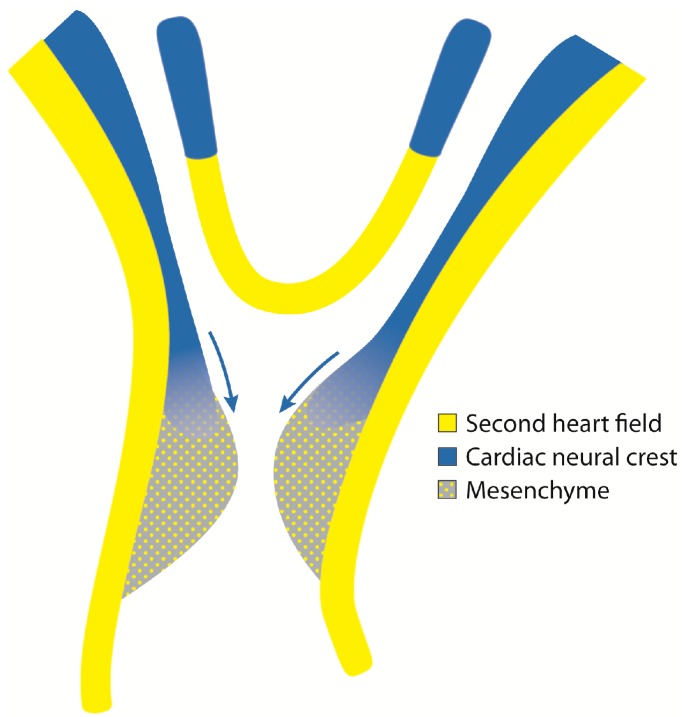
Remodeling of the endocardial cushions. Cardiac neural crest cells (blue) provide paracrine signals to second heart field cells (yellow) to orchestrate apoptosis (depicted as dark gray cells) and changes in extracellular matrix production during semilunar valve remodeling. Modified from [25].

Septation of the OFT requires formation of the truncal and conal cushions situated in the distal and proximal OFT, respectively. CNC cells form the mesenchyme of the truncal (right-superior and left-inferior) cushions that subsequently fuse to form the aortopulmonary septum, dividing the distal OFT into the aorta and pulmonary trunk [7]. Separation of the aortic and pulmonary trunk (aorto-pulmonary septation) occurs when the distal portions of the truncus swellings gradually fuse together in a cranial to caudal direction, to septation. By day 23–25 of human embryogenesis, the heart tube is formed and anterograde circulation begins, with development of the arterial pole over the next 5 days [20]. Septation of the aorto-pulmonary trunk results in the formation of the definitive intrapericardial pulmonary and aortic outflow channels, each of which possesses three segments, namely, the intrapericardial arterial trunks distal to the sinotubular junction, the arterial roots with their valves, and the subvalvar ventricular OFT. Separation of the aorta and pulmonary artery is completed by expansion of the aorto-pulmonary septum into the mesenchymal tissue of the proximal truncus swelling, separating the arterial valves (Figure 4). Mouse and chick studies have shown that abnormal EMT resulting in either deficient or excessive OFT cushion formation can give rise to structural OFT defects [36].

### 3.2. Development of the Aortic and Pulmonary Valves

The boundary at the dog-leg bend in the OFT identifies the border of the primordial OFT destined to become the sinotubular junction [44]. The valves and their supporting sinuses develop from the proximal part of the tract. Aortic and pulmonary valve development begins at approximately days 31–35 in humans, from the endocardial cushions in the OFT and atrioventricular canal of the primitive heart tube [45]. In early stages of arterial valve development, endothelial cells overlying the primitive endocardial cushions invade the cushion matrix, resulting in relatively bulky and cellularized endocardial cushions [46].

**Figure 5:**
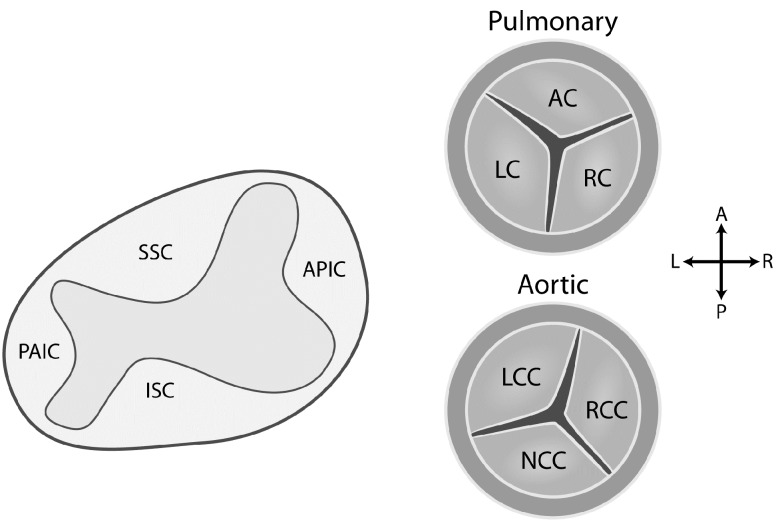
Development of the leaflets of the aortic and pulmonary valves. The semilunar valves arise from the conotruncal and intercalated cushions of the outflow tract. The conotruncal (superior and inferior septal) cushions give rise to the right and left leaflets of each of the semilunar valves. In the aorta, these are the right and left coronary leaflets, while in the pulmonary valve, these are the right and left cusps. The right-posterior and the left-anterior intercalated cushions develop respectively into the posterior aortic (non-coronary cusp of the aortic valve) and the anterior pulmonic (anterior cusp of the pulmonic valve) leaflets. Abbreviations: AL, anterior leaflet; APIC, anterior pulmonary intercalated cushion; CA, coronary artery; ISC, inferior septal cushion; LL, left leaflet; LCL, left coronary leaflet; NCL, non-coronary leaflet; PAIC, posterior aortic intercalated cushion; RCL, right coronary leaflet, RL, right leaflet

The semilunar valves arise from the conotruncal and intercalated cushions of the OFT, with the conotruncal cushions give rise to the right and left leaflets of each of the valves. In the aorta, these are the right and left coronary leaflets, while in the pulmonary valve, these are the right and left cusps (Figure 5). From the opposite quadrants of the OFT, the right-posterior and the left-anterior intercalated cushions develop respectively into the posterior aortic (non-coronary cusp) and the anterior pulmonic (anterior cusp) leaflets. These semilunar valve leaflets also derive their mesenchyme primarily from the endocardium [47]. The muscular wall of the proximal OFT does not have a cellular contribution to the valve leaflets or their supporting arterial sinuses, but does contribute to their growth in paracrine fashion. The rudimentary arterial valve leaflets and sinuses are formed by creating “cavities” in the fused distal parts of the proximal endocardial cushions, along with similar cavities in the new cushions through apoptosis and alterations in the extracellular matrix (Figure 5) [48]. Cavitation of the cushions results in a central lumen of each cushion that separates the three valve leaflets, with the peripheral portion arterializing to form the wall of the supporting valve sinuses. Valvulogenesis continues with elongation and thinning of the endocardial cushions by remodeling and compartmentalizing into the collagen-rich fibrosa, proteoglycan-rich spongiosa, and elastin-rich atrialis/ventricularis layers [46]. The extracellular matrix composition and organization of the valve leaflets are critical for normal valve function, and dysregulation of extracellular matrix remodeling or structural components can lead to valve malformations [49].

## 4. Transcriptional Regulation of Valvulogenesis

**Figure 6:**
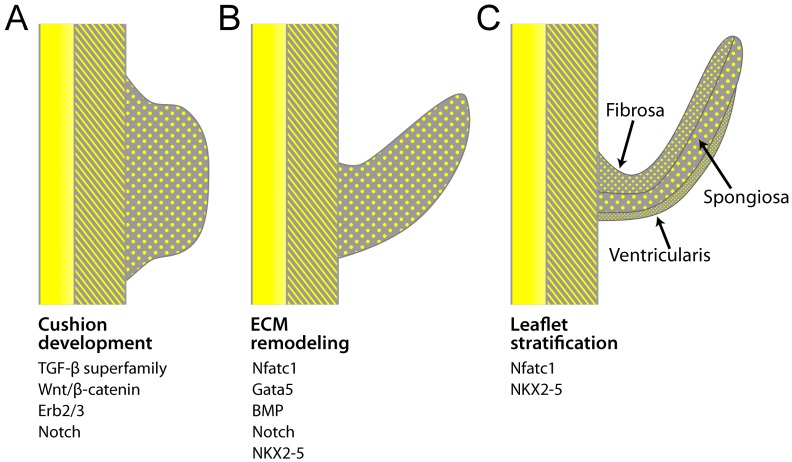
Transcription factors active in aortic valve development. (**A**) Endocardial cushions (purple) form in the cardiac outflow tract. Valve progenitor cells are generated by an endothelial-to-mesenchymal transition (EMT); (**B**) Endocardial cushions elongate to form valve primordia of individual valve leaflets. Extracellular matrix (ECM) remodeling and morphogenesis of the valve leaflets is dependent on Nfatc1 and Gata5; (**C**) ECM of the primordial semilunar valve thins, elongates and stratifies into fibrosa, spongiosa, and ventricularis layers. Modified from [45].

The gene regulatory network of valve progenitor cells in the endocardial cushions includes the complex spatial and temporal interplay of transcription factors involved in EMT and mesenchymal progenitor populations [50]. Endocardial cushion formation is initiated by signals from the OFT myocardium that cause adjacent endocardial cells to undergo EMT. Many transcription factors and signaling pathways have been implicated in EMT and cushion morphogenesis, including members of the TGF-β superfamily, Notch, BMP and Gata families, Nfatc1, Wnt/β-catenin, Twist-1, Sox9 and others (Figure 6). The complexity of cardiac structure and functioning cells types developed from an apparently few embryonic cell types is surprising. Similarly, the marked localization of single-gene defects to specific cardiac structures appears at odds with the presence of many of these proteins in numerous cell types of the heart and other organs [5].

Ascertaining the timing and relative expression of these transcription factors is difficult and has been complicated by misinterpretation of results from older studies using less robust techniques and non-human models. Nevertheless, an expanding list of transcription factors appear to have important roles, or at least are well described. These factors overlap with genes that have been implicated in BAV, but to a limited extent.

### 4.1. Notch

Signaling factors, particularly from the Notch and TGF-β families, facilitate EMT and mesenchymal invasion of the OFT cushions [51]. The Notch pathway is involved in the majority of cardiac embryogenesis, notably including valve development [52]. *Notch1, 2* and *4* receptors and their ligands (Jag1/2 and Dll4) are expressed in the OFT and cushions throughout valvulogenesis [51,53,54,55]. When activated, the Notch intracellular domain (*NICD*) translocates to the nucleus where it creates a complex of *NICD*, *Rbpj*, and the inhibitor *MAML* leading to activation of *Notch* target genes. In the embryonic valve, *HES*, *HEY*, *Acta2*, *Snai2*, *Smad3*, and *Runx3* are direct Notch target genes [56]. Notch activation also inhibits TGF-β-induced *Smad1* and *Smad2* signaling, subsequently decreasing expression of their target genes [57]. Underscoring the importance of *NOTCH*, its inactivation or mutation leads to multiple forms of CHD, such as BAV [15], Alagille syndrome [58,59] and hypocellular endocardial cushions [57,60].

Several mouse models have been generated for *Notch1/2/4* and their downstream effectors to examine the functions of Notch signaling during valve development. In mice, *Notch1/2/4*, *Rbpj* and *Maml* knockouts are embryonic lethal, with affected embryos having OFT and other cardiac and non-cardiac abnormalities [51,57,61], whereas *Notch3* knockouts do not have an abnormal phenotype [62]. Demonstrating the complexity of pathways in aortic disease, haploinsufficiency of *Notch1* on a mouse *NOS3^−/−^* background causes marked aortic phenotypes, whereas each mutation alone has very limited phenotypic changes [63,64,65]. Interestingly, single-gene knockouts of the cardiac ligands of *Notch*—*Jag1*, *Jag2* and *Dll4*—also do not show valve abnormalities, perhaps indicating redundant signaling. Importantly, Notch has been implicated in mouse and human calcific aortic valve disease (CAVD), a common form of heart valve disease [66,67,68].

### 4.2. BMPs and TGF-β

BMPs 2–7 and TGF-βs are part of the TGF-β superfamily expressed in the embryonic heart. BMPs bind to cardiac BMP Type I receptors—ALK2 (activin-like kinase) and ALK3—and Type II receptors—BMPRII, ActRII or ActRIIB—that activate an intracellular canonical signaling pathway mediated by Smad4 [69,70]. Similarly, three TGF-β ligands (TGF-β 1/2/3) bind to cardiac Type I (ALK5) and Type II (TGFBRII) receptors to activate canonical TGF-β signaling [69] mediated by Smad2 and Smad3. This transcriptional pathway in conjunction with Smad inhibitory proteins regulates transcription of TGF-β superfamily-responsive genes [71]. BMP, Notch and TGF-β promote EMT, mesenchymal cell invasion into the cardiac cushions and remodeling of the valves [69,72]. Specific to formation of the arterial valves, TGF-β signaling plays an essential role in the initial promotion and cessation of EMT, and in cushion mesenchyme proliferation and in differentiation during heart valve development.

BMP, TGF-β and Notch are not only activators of these processes, but also transcriptional repressors via the *Snail* family of proteins that are required for the initial steps in EMT and cushion development [73]. As *BMP, TGF-*β and *Notch* all target *Snail* expression, Snail is likely a critical point of convergence for their signaling in valve development. In the adult heart, abnormal BMP signaling has been found in CAVD [74,75], implicating it in the pathogenesis of this condition. Numerous cardiac tissue–specific knock-down experiments have established the importance of the BMP pathway in the development of the endocardial cushions and valve development [76,77,78]. In contrast, cardiac tissue–specific knock-down of TGF-β1 yields little structural defect. Knock-down of TGF-β2 does produce structural OFT, septal and aortic arch defects [79,80], along with hypercellular cardiac cushions and valves [81]. Important to the initiation and progression of CAVD, TGF-β signaling is involved in the activation of valvular interstitial cells and their transformation to myofibroblasts in the adult valve [82].

### 4.3. Gata

The *Gata* family of zinc-finger transcription factors binds the “GATA” nucleotide motif and plays an essential role in cardiac development [83]. With relevance to the aortic valve, *Gata4* and *Gata5* are expressed in endocardial cells, endocardial cushions and OFT, and *Gata6* is expressed in CNC cells [84]. Abnormalities in mice with *GATA* mutations indicate that interaction between *Gata4* and *Gata5* is necessary for the endocardial cushions to develop, as *GATA4*^+/−^ *GATA5*^+/−^ double heterozygotes have enlarged semilunar valves compared with single *GATA4*^+/−^ and *GATA5*^+/−^ heterozygotes [84]. *Gata4* mutant mice and humans show septal defects, including both atrial and ventricular septal defects [8,85,86,87,88].

### 4.4. Nuclear Factor in Activated T-Cell, Cytoplasmic 1

In the embryonic heart, *Nfatc1* is expressed before EMT of the OFTs and is seen in endocardial cells, but not in transformed mesenchymal cells of the endocardial cushions [41]. It is also expressed after rudimentary valve formation, enabling elongation of the valve leaflets through degradation of the extracellular matrix of the endocardial cushions by enzymes such as cathepsin K [89]. *Nfatc1*-deficient (*nfatc1*^−/−^) mice have normal endocardial cushion formation but fail to begin normal remodeling of the endocardial cushion for arterial valve development, and this deficiency is embryonic lethal [90].

In addition to its role in valve development, *Nfatc1* also controls NFκB ligand (*RANKL*), a member of the tumor necrosis factor ligand family that is a key factor for osteoclastogenesis and that signals through its receptor *RANK* to promote differentiation of bone-resorbing osteoclasts [91]. The transcription factor *Nfatc1* transduces signals from *RANKL* for differentiation of osteoclast-mediated bone resorption [91] by controlling expression of β3-integrin [92]. Thus, common defects involving *Nfatc1* may be at play in BAV, CAVD and ascending aortic aneurysm. 

### 4.5. Nkx2-5

The homeodomain factor *NKX2-5* is the most commonly mutated single gene in human CHD, accounting for 1%–4% of specific malformations, including cardiac conduction and OFT abnormalities as well as atrial and ventricular septal defects. However, lack of mutational genotype-phenotype correlation points to an early role for this factor in development of the FHF and SHF, and possible phenotypic modification by downstream genes [93]. Although *Nkx2-5* is widely expressed in the developing heart, its most significant role appears to be in SHF development, specifically, directing SHF specification and morphogenesis of SHF-derived cardiac structures [94,95]. *Nkx2*-5 represses *Bmp2*/*Smad1* signaling and regulates SHF proliferation and OFT morphology [94], and appears to require *Mef2c* for its action [96]. However, for a gene associated with so much human CHD, we know little about its mechanisms of action in cardiac development, especially considering its importance in late natal and post-natal cardiac development [97].

### 4.6. Nitric Oxide

The first non-human BAV was observed in *NOS3* deficient mice, where the normal expression of *NOS3* expressed in aortic valve endothelium of adult mice was not observed [63]. Structural cardiovascular abnormalities of the aorta were not observed in *NOS3^−/−^* mice except when combined with haploinsufficiency of *NOTCH1* [65]. The role of *NOS3* in adult development of aortic aneurysm, coarctation or aortic valve stenosis is not clear as aortic wall NOS expression is lower in bicuspid aortic aneurysm [98] and in the aortic root [99] but not associated with aortic stenosis or aneurysm in *NOS3^−/−^* mice [100].

## 5. Etiology of BAV Disease

The mature aortic valve has cusps composed of three overlapping layers of highly organized extracellular matrix, embedded with valve interstitial cells and covered by a layer of valve endothelial cells [101]. The three layers are the fibrosa, spongiosa and ventricularis (Figure 6). The fibrosa, which is located on the arterial aspect of arterial valves, is composed predominantly of fibrillar collagens (Types I and III) that are circumferentially oriented and provide tensile stiffness [46,47,48,49]. The ventricularis layer, located on the ventricular side of the arterial valves, is composed primarily of radially oriented elastic fibers that facilitate tissue motion [50,51]. The middle layer, the spongiosa, is composed primarily of proteoglycans with interspersed collagen fibers. Proteoglycans, which are also present in the other layers, serve as an interface between the orthogonally arranged fibrosa and ventricularis layers to provide tissue compressibility and integrity.

Like other forms of congenital heart disease (CHD), although chromosomal (DiGeorge and Turner syndrome) and Mendelian (*NOTCH1*) causes of BAV have been identified, these account for a small percentage of BAV cases [102]. The genetic mechanisms underlying the majority of “sporadic” cases of BAV are not known. Even for these seemingly sporadic cases, epidemiological studies have demonstrated a roughly 10% increased risk of BAV in siblings and offspring, and a similar figure for the occurrence of aortic aneurysm in relatives with, or without, BAV, indicating the potential role of shared genes or even environmental causes [103,104].

The genetic architecture of sporadic BAV likely includes: (i) accumulation of rare and uncommon variants in cardiac developmental genes leading to a mutational load favoring BAV formation; (ii) epigenetic modification of cardiac developmental genes; (iii) common variants in genes that may not be obviously linked to cardiac development but have impact on their function; and perhaps; (iv) environmental causes. Complicating this scenario, many CHD-causing gene variants are likely selected against by a decrease in reproductive fitness. The resulting allelic heterogeneity reduces the power of GWAS for CHD. This may or may not be an issue for BAV, as its effect on reproductive fitness is thought to be limited. Nevertheless, these issues collectively make the discovery of the etiology of BAV difficult (Table 1).

**Table 1:** Factors that may confound discovery of BAV-associated genes.

Low frequency of individual variants causing BAV
High number of variants causing BAV
Variants of many structural types
Variants of many functional roles
Mixed inheritance patterns of disease
High fetal loss of CHD

BAV is commonly associated with aneurysm of the aortic sinuses, the ascending aorta, and aortic coarctation. Pathological examination of the BAV-associated aortic aneurysm often shows non-inflammatory degeneration of smooth muscle cells often described as cystic medial necrosis [105,106,107]. The co-occurrence of BAV with aortic aneurysm and coarctation of the aorta may imply a common genetic mechanism for these diseases. Dysregulation of the canonical TGF-β signaling axis in humans offers one such example. As described above, TGF-β signaling is a key pathway promoting EMT in valvulogenesis and cellular migration, activities critical to normal valvular development. Mutations in the canonical TGF-β signaling pathway may therefore be hypothesized to cause an enhanced rate of BAV. The human condition Loeys-Dietz syndrome (LDS) is caused by mutations in the genes encoding the TGF-β receptors 1 and 2 (*TGFBR1* and *TGFBR2*), the TGF-β ligands 2 and 3 (*TGFB2* and *TGFB3*), as well as in the downstream transducer of TGF-β signaling, SMAD3. BAV is frequently encountered in patients with LDS, as well as a nearly complete penetrance of thoracic aortic aneurysm [108,109]. While overt TGF-β dysregulation may be an uncommon mechanism for typical BAV-associated thoracic aortic aneurysm, minor variation in other pathways that overlap functionality between valvulogenesis and arterial development may be common in patients with BAV-associated thoracic aortic aneurysm. While one commonly encounters arguments that aortic aneurysms are solely the result of abnormal flow patterns around the valve, these arguments cannot properly account for observed individual variation and the lack of correlation between hemodynamic derangement and aneurysm size seen in BAV patients. Therefore it seems likely that genetic variation in addition to hemodynamic factors plays a major role in aneurysm susceptibility in typical BAV disease.

**Figure 7:**
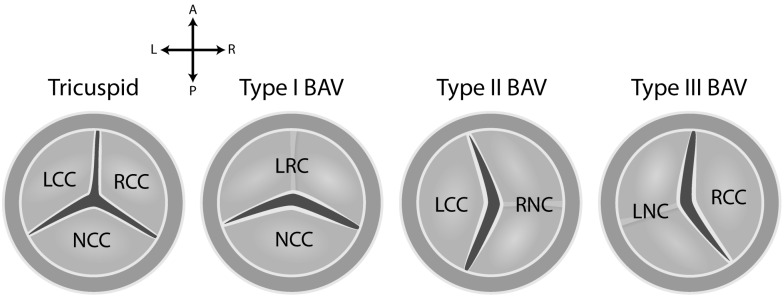
Anatomy of the bicuspid valve and potential pathways for its development. The bicuspid valve is classified as Type I: fusion of the right coronary cusp (RCC) and left coronary cusp (LCC) to create a fused left-right cusp (LRC). A Type I BAV results from persistent fusion of the left and right leaflets normally be formed by the superior and inferior septal cushions. Type II: fusion of the RCC and non-coronary cusp (NCC) to create a fused right-non-coronary cusp (RNC). Type III: fusion between the LCC and NCC to create a fused left-non-coronary cusp (LNC). Both Type II and Type III BAVs result from fusion of the posterior aortic interacted disc with either the inferior (Type II) or superior (Type III) septal cushion. These differences in anatomy imply differences in etiologic mechanisms [110].

As previously mentioned, BAV is the most common of congenital valvular defects and is characterized by a valve having only two commissures (Figure 7). It is commonly associated with aneurysm of the aortic sinuses and the ascending aorta, and aortic coarctation. Pathological examination of the BAV-associated aortic aneurysm often shows non-inflammatory degeneration of neural crest-derived smooth muscle cells often described as cystic medial necrosis [105,106,107]. Nevertheless, experimental evidence supporting a common underlying developmental mechanism to explain the association of aortic valve and associated aortopathy is lacking.

Human BAV has been associated with chromosomal variation in DiGeorge syndrome (22q11.2^del^) and Turner syndrome (Xp^del^). The genetic basis of structural valve abnormalities in DiGeorge syndrome has been attributed to hemizygosity of *TBX1* and impaired downstream signaling by *Fgf8* [111,112], but the association is imperfect. The cause of BAV, often with coarctation, in 30% of women with Turner syndrome is unknown, although good evidence has implicated a haploinsufficient region lying distal to Xp11.4 [113,114,115]. The very high incidence of BAV in Turner women compared with XY men favors existence of autosomal modifier genes for this valvular disease and perhaps a homologous Y chromosomal gene [4]. BAV has also been associated with the primary ciliopathies, such as Joubert syndrome [116], and with ventricular non-compaction [117,118]. Using linkage, loci within the 5q15-21, 13q33-qter and 18q22.1 chromosomal regions have been identified [4,119], but the responsible genes have not.

Smaller abnormalities such as copy number variation, indels and single-nucleotide polymorphisms almost certainly have some responsibility for BAV in the general population, especially in sporadic cases not associated with extensive non-valvular phenotypes. Variants in *NKX2-5* (5q34) [120,121], *NOTCH1* (9q34.3) [15,16,122,123], *FBN1* (*15q21.1*) [124], *MATR3* (*5q31.2*) [125] and *GATA5* (20q13.33) [126,127,128] have been observed in individuals with BAV. However, some of these associations may result from co-existing disease, such as association of FBN1 with ascending aortic disease [129]. Unfortunately, none of these findings provide epiphanous insight into the causes of BAV.

Study of the cause(s) of BAV is a good example of the limitations of disease mapping using genetic variation to explore human complex disease (Table 1). There may be significant cost and time advantages to discerning the cause of BAV and its co-existing diseases using vertebrate models of the condition. Despite limitations in animal models of human disease, targeted “knock-in” candidate gene mutations in mice [130] and zebrafish [131] may provide useful insight by modeling human BAV better than gene knockout mouse models do [130]. These individual SNPs or small insertion/deletion mutations are far more frequently observed in human disease than whole-exome or whole-gene deletions. In addition, these smaller variants more accurately replicate human disease and thus have greater potential applicability. The value of large or whole-gene deletion is principally confined to identifying pathways of disease rather than the exact molecular biology of disease. Although these techniques are a necessary albeit limited step, they cannot explain biological functions of the candidate genes identified. Culturing of embryonic and aortic valve interstitial cells will be needed to validate these targets and identify their relationships to subsequent CAVD and aortic disease [3,130].

## 6. Summary

In this review, we have described the series of events from cellular and transcriptional embryogenesis of the heart, to development of the aortic valve. The frequent occurrence of BAV and its anatomically discrete but common co-existing diseases (ascending aortic disease, aortic stenosis and coarctation of the aorta) leads us to suspect a shared cellular origin. However, the extensive cell-cell signaling and interactions of cardiac embryogenesis preclude simplistic explanations of this disease.

## Abbreviations

ALK = activin-like kinase; BAV = bicuspid aortic valve; BMP = bone morphogenic pathway; CAVD = calcific aortic valve disease; CHD = congenital heart disease; CNC = cardiac neural crest; EMT = epithelial to mesenchymal transition; FGF = fibroblast growth factor; FHF = first heart field; GWAS = genomewide association study; LPM = lateral plate mesoderm; Nfatc1 = nuclear factor in activated T-cell, cytoplasmic 1; NICD = Notch intracellular domain; OFT = outflow tract; SHF = second heart field; SNP = single nucleotide polymorphism; TGF-β = transforming growth factor-β; Wnt = wingless-related MMTV integration sites.
